# Monthly Summary

**Published:** 1883-08

**Authors:** 


### MONTHLY SUMMARY.

---

THINGS TO BE CONSIDERED IN REGULATING TEETH.—In regulating teeth, that is in bringing them from an abnormal into a normal position in the arch, several things have to be taken into consideration. First, we must devise an appliance that will be effective; second, it must be constructed as simply as possible, third, it must do its work as quickly as possible consistent with the existing circumstances; fourth, it should give as little pain as possible; and lastly, it must be fixed in its position, and not liable to slip and cause injury and possibly defeat its object. Some of these considerations are too frequently over-looked by many who undertake this difficult class of work. There have been many systems, so-called, for the correction of irregularities, but no one of them contains all that is good or all that we need. The successful practitioner in this line is he who takes from each "system" that which will best serve his purpose, and then uses with discretion. We sometimes see appliances for regulating, illustrated and recommended in our journals, that would require a master mechanic to construct and a skilled engineer to run. The idea also of moving teeth only on exact mathematical distance in each twenty-four hours, is a fallacy. What we want is to use appliances not too powerful, and then push our work along as rapidly as possible, having regard, of course, to the age and health of patient and the surrounding conditions. Much harm has been done in years past by the slipping of appliances and the consequent wounding or injury of the soft tissues. The pain that is usually attributed to the moving of the teeth too rapidly, I have satisfied myself, is due to the rubber band, or ligature, or plate, or appliance, impinging upon the gum. To avoid this, I have for the past ten years adopted a plan of keeping all my fixtures from touching the soft parts. I simply make bands or rings of platinum plate, and slip one over the tooth to be moved and the other over the one that I want to use as my fixed point of resistance. To these have been previously soldered either pins, hooks, or bars, as the case may be. The bands are lined with phosphate of zinc and slipped over their respective teeth to a point about midway between the cutting-edge and neck. Being kept dry for five minutes to harden, they will afterward resist all the force we may apply to them without becoming loosened. Out of scores that I have applied in this way, none that were properly placed have ever become loosened, though force has been applied to them for months continuously. If we wish to use a jack-screw as our power, instead of a rubber band or bar, we need only solder stays or abutments to these bands at proper places and counter-sink them to receive the ends of the jack-screw. By their use we will have no slipping of fixtures, no impinging upon gum, and hence the very least amount of pain. In a couple of cases recently treated in this way, in each of which a central incisor was moved the width of its crown, and moved, too, as rapidly as double rubber rings would move it, occupying about a month's time in each instance, both patients have declared to me time and again, that they suffered absolutely no pain.—*Dr. Guilford, Dental Cosmos.*

BE TOLERANT.—In perusing dental literature as we find it in the journals of the period, one is astonished at the lack of respect often shown to one who expresses an honest opinion on any subject connected with dental practice. Instead of carefully weighing statements upon disputed points, and giving them careful consideration, they are, without argument and investigation, pronounced incorrect, according as the critics' former opinion—or oftener, prejudice—leads. Especially if opinions are published that are at variance with long accustomed practice, do we find this intolerance manifested.

No sooner does an investigator publish the result of long and close study, than he is met with somebody's saying "It is not so;" and his objector will not even take the trouble to state his reasons, satisfied to denominate all of his opponent's logic as "meaningless rhapsodies" or "senseless declarations," and then proceed to state his own side of the case, to be met with his own weapons when the first speaker gains the ear of the public.

Antagonism in science will often be the surest means of reaching the truth, but it must be an antagonism dignified with argument, that gives reasons for its statements, and allows due weight to the opposition, not an ignorant intolerance that decries everything and everybody if they oppose preconceived notions. The dental writer or critic should bear in mind that an intolerant person is generally either ignorant or ill-bred, and always give deference to statements that are the result of thought, labor, and experience. In each case where there is a difference of opinion in dental practice, it is very possible that seemingly incontrovertible reasons have lead the disputants to their different opinions, and so we are lead to see that many circumstances in one case may give color to a statement, that are wanting when an attempt to prove that statement is made in other hands.

It is possible, while every man's ability and cultivation is different from that of its neighbor—none identical—that opposite statements will not be made upon an infinite variety of subjects; and so we plead for tolerance, and believe that as a rule, the man whose ability and education ranks high, is the man who gives civil answer to those differing in opinion with him.—*Editorial in Missouri Dental Journal.*

TEMPERING STEEL.—More tools are ruined by over-heating, cold-hammering and over-tempering, says the *Scientific American*, than can be redeemed by all the new recipes that have been invented. The only way that really good is first to find a brand of steel that is good and suitable for the tools to be made, and stick to it. Next find by a few trials the lowest heat that will harden it in pure water at 70°, or ordinary shop temperature. If steel is hardened at the lowest heat the temper will require drawing very little, that is, to a pale straw, full straw, or brownish yellow, but not deeper unless for wood-working tools with thin cutting edges, when a full brown may be desirable. File-makers use salt water for a hardening bath, because it makes the water more dense and the teeth harder, and of course more brittle. Sulphuric acid or mercury is sometimes used for hardening very small tools for cutting glass and etching stone. For springs the same care should be taken in regard to low even heating that is necessary with tools. Pure lard oil is as good, and probably better than any of the many mixtures that have been tried for the hardening fluid ; burning off may do for drawing the temper of small or thick springs, but is totally unfit for long or slender ones. Dip the hardened spring into a bath of oil heated nearly to its boiling temperature ; this is the only way to get an even temper.

In case a "*barbe*" is broken off and fixed in the pulp canal of a tooth, so that it is impossible to remove it by mechanical means, pack the cavity with common salt, seal with "Hill's Stopping," and let it remain two or three days, when the "barbe" will be found to have become an *oxide* and can be easily removed by thoroughly syringing the cavity with water. —*New England Journal of Dentistry*.

---

MANKIND'S MISTAKES.—It is a mistake to labor when you are not in a fit condition to do so.

To think that the more a person eats the healthier and stronger he will become.

To go to bed at midnight and rise at daybreak and imagine that every hour taken from sleep is an hour gained.

To imagine that if a little work or exercise is good, violent or prolonged exercise is better.

To conclude that the smallest room in the house is large enough to sleep in.

To eat as if you only had a minute to finish the meal in, or to eat without an appetite, or continue after it has has been satisfied, merely to satisfy the taste.

To believe that children can do as much work as grown people, and that the more hours they study the more they learn.

To imagine that whatever remedy causes one to feel immediately better (as alcoholic stimulants) is good for the system, with regard to the after effects.

To take off proper clothing out of season, simply because you have become heated.

To sleep exposed to a direct draught in any season.

To think that any nostrum or patent medicine is a specific for all the diseases flesh is heir to.—*Index.*

---

A RARE CASE OF EXOSTOSIS.—On the 21st of March, 1883, Mrs. B., aged 56, called at my office for the purpose of having the remaining eight superior and one inferior tooth extracted, preparatory to having artificial sets inserted, they being badly decayed. The first tooth I extracted was the right superior, lateral incisor, which required a great deal of force to remove it. I found a square shoulder formed, about half way from the neck to the apex, the enlargement extending to the apex, making the exostosed part fully one-half larger in diameter than the part above. I then removed the first bi-cuspid, on the same side. I found the same condition of osseous formation. I then removed the canines, and found them in the same condition. I then removed the first and second molars, on either side. The roots were nearly obliterated by the osseous deposit. The lady informed me that all her other teeth had been in the same condition. I have, at different times, in a practice of ten years, removed as many as two teeth in the same mouth that were exostosed. I do not remember reading of a case where the entire denture was exostosed. This may not be a rare case, but I thought it might be well to present it, if not for the information of my confreres, for their opinions. The lady said she had never suffered very much with her teeth, but had been a great sufferer for several years, from neuralgic pains in her eyes and forehead, with quite a weakening of the optic nerve. These pains were not periodical, as is usual, but constant.

Is this neuralgia, and the partial loss of sight, a sequence of the exostosed condition of the teeth ? I shall watch the case with interest, and expect an improvement in the eyesight, and the disappearance of the pains in the forehead.—*Dr. L. M. Mathews, of Fort Scott, Kansas, in Ohio Journal.*

---

THE EFFECT OF NITRATE OF SILVER ON THE SKIN AND MUCOUS MEMBRANE.—Mr. J. S. F. æt. 33 years, of Columbus, Mo. At the age of about fifteen years he took from five to ten drops of a solution of nitrate of silver of the strength of grs. xx ad. ℥ j. This was continued for about five or six months ; at the end of this time he noticed that his face and hands were getting a peculiar dark color. The color increased for some time after he discontinued taking the siver solution.

The color of the integument of his face and hands at the present time resembles a No. 2 lead pencil mark, with a light sky-blue in it. A stove merchant, who happened to see him in my office, thought that his face and hands had the appearonce of being colored with a light coating of stove polish, which is really a very good description of his appearance, as the skin of his face, especially, has a marked polished appearance, or a shine to it. The color is decreased during cold and dry weather and increased during damp and hot days.

The mucous membrane lining the anterior nares, the inside cf the mouth, the lips, the under portion of the portion of the tongue, the soft palate tonsils, pharyngo-nasal cavity, larynx and vocal cords were all colored by the nitrate of silver, so were also the membrana tympana and the scherotic coat of the eyes.

The whole of his body was more or less colored, but not to so marked a degree.—*St. Louis Med. and Surg. Journal.*

---

EAR-ACHE IN CHLIDREN.—Dr. Sam'l Sexton contributes an article on this subject to the *Medical Record,* May 5, 1883, in which he deprecates routine treatment or injections until we have found out the cause of the ache by careful examination, for in a very large number of the ear-aches in childhood the causes are to be sought elsewhere than in the hearing organ itself, and they will be found to depend, for the most part, on nervous sympathy; the most prominent are dentition, dental caries, and clods in the head. Thus nervous impulses propagated from regions remote from the ear may give rise to pains in the ear—neuralgic otalgia—without perceptible hyperæmia; or the intensity of the congestion arising in a part so richly supplied with blood-vessels may manifest itself as an acute aural catarrh.—*Med. and Surg. Reporter.*

---

HARDENING STEEL.—According to a Sheffield paper a very fine preparation for making steel very hard is composed of wheat flour, salt, and water, using say two teaspoonsful of water, one half a teaspoonful of flour, and one of salt. Heat the steel to be hardened enough to coat it with the paste by immersing it in the compound, after which heat it to a cherry red and plunge it into soft water. If properly done the steel will come out with a beautiful white surface. It is said that Stubbs' files are hardened in this manner.—*Druggist's Circular.*

---

GLUTEN.—Flour deprived of the *gluten* of the wheat, under which general name may be classed the phosphatic and nitrogenous elements—which are stored principally between the outer wraps and the inner starch body of the kernel—has lost the greater part of its blood-making materials. This gluten of wheat may be compared to the lean of meat, while the whiter starchy portions of the wheat may be compared to the fat of meat.—*American Miller.*

---

PRACTICAL SUGGESTIONS DURING DENTITION.—This is an important subject, and one that should interest every dental and medical practitioner, and also parents, or any that have children under their care. They should be instructed in this important branch ; and children, during the period of dentition, should be under the care of an intelligent instructor. The mother during pregnancy, and especially after fœtal life, while the child is receiving nourishment from her, should live according to the laws of nature, eating suitable food to sustain the different elements that compose the osseous structures. A child must have food to build up the system instead of tearing it down, if we wish to improve the present as well as the future generations.

Dentists should use their influence in this great and important work. Parents are often ignorant of the laws of nature, and deprive their children of nourishing food.

How could we expect a tree to grow and bring forth fruit, if deprived of the elements that nature provides for it? Deprive it of the sun, the showers, and rich soil, and it will suffer. The same with the child ; if certain laws are ignored it will grow up weak and delicate, and the teeth, in such cases, are apt to be in accordance with the body. Ignorance is frequently the cause of suffering. Children are often brought into existence, and suffer from the neglect of others, and are deprived of the proper nourishment that nature provides. Our profession as a body seems to ignore this great and important subject, which should claim the attention of every intelligent dentist.

A child should have our-door exercise, regular baths, and during the winter months should be well dressed, so as not to be influenced by sudden changes. And the most important of all, it should eat suitable food that will build up the system, and thus the present generation can be greatly improved. We have had marked success in this direction in many cases in the past few years. I will simply mention one for illustration. A child three years old was brought under my care for treatment. I found the teeth in a bad condition ; the labial surfaces of the superior teeth were wasting away, were very soft and sensitive, saliva in an acid condition. I filled the cavities or surfaces with a phosphate preparation, and found, by properly questioning the parents, that the child had been deprived of suitable food ; that its diet was principally sweatmeats, and it was in the habit of eating between meals, such as pie, cakes, etc.; and at meal-time, when the family had substantial food, the child seldom ate. I advised a change of diet, and if piece-meals were necessary, give it substantial food, such as beef, eggs, potatoes, milk, wheat, corn, and Graham bread. The diet wes changed at once, and proper exercise was rcommended. The child's general health was greatly improved. Two years have passed, the teeth are quite firm, and solid in structure, and no doubt all can be preserved till nature sees fit to throw them off to make room for the permanent teeth, which will be improved by strictly following the iaws of hygiene.

Medical men might have much influence in this direction if they would turn their attention that way; but they neglect the matter, and it is simply left with us for correction.—*Ohio Journal.*

